# Modern Sociology

**Published:** 1899-04-01

**Authors:** 


### Modern Sociology.

### THE GIRL FROM THE UNION.

Workhouse life is bad for a child, and perhaps the worst part of it is that it must one day end, and that the child accustomed to have everything done for him, his actions and even his thoughts controlled, is suddenly thrust into the battle of life with almost no knowledge of the world and its ways. It is for this reason that we have always advocated boarding-out, with all its difficulties, and the risk of the boarded-out child being treated harshly by its foster-parents. At least it gives that knowledge of good and evil which, now that we are hopelessly out of Eden, is an essential part of the education of every mortal creature. But it is practically impossible for large unions to find suitable foster-homes for all their children, and it is equally impossible to keep them under the surveillance of the parish authorities all their life. Independence must come some day with all its dangers, and all that can be done is to see that the little pauper children start life under as good auspices as possible.

The risks that attend boys are great enough, but perhaps they are not as liable to come to swift and hopeless shipwreck as girls: A girl of thirteen, sent out into the world just when she needs most to have a wise and kindly friend to advise her, is very apt to get into trouble. In such situations in domestic service as are open to her youth and ignorance, the work is often hard, the conditions disagreeable, and the mistress (often with the best intentions) severe; and at hand, when an hour of freedom can be snatched, are temptations whose dangers the child cannot know. She has had less than her fair share of pleasure hitherto, she is not freely given any now, and she grasps at what she can, not knowing whether it is wholesome or the reverse. She falls in with bad companions, vanity may lead her into dishonesty, and when temptation comes in the guise of love, the natural impulses of the poor, starved young heart may lead her to hopeless ruin.

There is here, we think, a large field for ladies who really want to help such girls. But they must first take to heart the fact that a girl from the union is just like other girls—only more so. She will be more fond of dress than the average, because she has always worn a dreary uniform. She will be more eager for sweets, and shows, and penny novelettes, than girls who have enjoyed some of these luxuries at home, before they went into service; and to blame her for this is absurd. Take the average girl of any class between the ages of fourteen and twenty and you will find that her mind is taken up with dress and amusement and love-fancies, often of the silliest and most unreal nature. And the girl from the union is just like her neighbours. To accept these tendencies as natural and not unwholesome, to give them moderate and reasonable indulgence, and thus keep them under control, is, in most cases, the first step in training a girl to be a good and useful woman; and with such treatment she may develop into a faithful servant and a loyal friend. The blunder made by many well-intentioned philanthropic agencies is the omission of pleasure from their scheme; but no agency can do so much for a girl in this way, and so well watch the effect of occasional indulgence as a wise and kindly mistress, who feels that she is, in very deed, *in loco parentis* to her servant.

Failing this, which cannot always be obtained, we have several associations which do admirable work in keeping in touch with young servant girls. There is, for example, the Metropolitan Association for Befriending Young Servants—the M.A.B.Y.S., as it is called in brief. The main work of this association is the care and guidance of girls who leave poor-law schools for domestic service and other young girls who are friendless. The association has five training homes in London and the neighbourhood, where the girls are taught laundry work and other domestic duties. It is a great matter for the girls thus to receive systematic training in their work. Many have remained all their lives poor ill-paid, ill-treated "slaveys" because no one ever took the trouble to teach them how to do their work well. Domestic work, when well done, wins good pay and many privileges but an incompetent servant is one of the most miserable of drudges. Besides training the girls in the first place, the association has provided 14 lodging-homes, where girls can stay when out of place, thus obviating the necessity of their returning to the workhouse for temporary shelter, or staying in such undesirable lodging-houses as they can afford. Ladies belonging to the association also visit the girls in their situations, and see if they are as safe and comfortable as it is desirable they should be. Real and permanent friendships are often formed thus, which exist long after the girl has left her first place, and perhaps has gone to the country or to some other town.

Similar work is done in the country districts by the Girls' Friendly Society, though that does not by any means confine its work to girls from poor-law schools, and this is in itself a good thing; for it is not desirable that a girl should go all through her life with the feeling that she is a girl from the union. The workhouse should pass into the background of her thoughts, as it passes into the background of her life; for its influence, even if not actually degrading, tends to maintain a notion of dependence which it is to be wished she should get over. On the other hand, it is something of a disadvantage that the Girls' Friendly Society is a voluntary one. Girls join it of their own free will, though, of course, wise advice may induce them to do so; and they have to pay a small subscription. But it does not, as a matter of course, look after all young girls in the places where it has branches. In small places the entertainments given by the society attract girls; but, in the larger towns, these can hardly compete with the ordinary places of amusement. Nevertheless, the society is doing a great deal of very good work.

It must be owned that there are mistresses who object strongly to their servants belonging to either association. Sometimes this is from a selfish fear of interference with the manner in which they treat them; but sometimes the objection is due to the way in which the lady associates of the society fulfil their task of looking after the girl. We have heard of a lady calling to visit the mistress, who was a personal friend, and stopping in the hall to ask the maid if she was happy and well-treated. Such a lack of tact and good breeding is sure to hamper the success of the effort, and prejudice mistresses against associations which, in themselves, make for their comfort and well-being as much as for that of their servants.

